# Extended spectrum beta-lactamase mediated resistance in carriage and clinical gram-negative ESKAPE bacteria: a comparative study between a district and tertiary hospital in South Africa

**DOI:** 10.1186/s13756-018-0423-0

**Published:** 2018-11-14

**Authors:** Raspail Carrel Founou, Luria Leslie Founou, Sabiha Yusuf Essack

**Affiliations:** 1 Antimicrobial Research Unit, School of Health Sciences, College of Health Sciences, University of KwaZulu-Natal, Durban, 4000 South Africa; 2 Department of Clinical Microbiology, Centre of Expertise and Biological Diagnostic of Cameroon, (CEDBCAM), Yaoundé, Cameroon; 3 Department of Food Safety and Environmental Microbiology, Centre of Expertise and Biological Diagnostic of Cameroon, (CEDBCAM), Yaoundé, Cameroon

**Keywords:** Antibiotic resistance, ESKAPE bacteria, ESBLs, Carriage, Clonality, Hospitalized patients

## Abstract

**Background:**

Gram-negative ESKAPE bacteria are increasingly implicated in several difficult-to-treat infections in developed and developing countries. They are listed by the World Health Organization as resistant bacteria of critical priority in research.

**Objectives:**

To determine the risk factors, prevalence, phenotypic profiles, genetic diversity and clonal relatedness of extended-spectrum β-lactamase (ESBL)-producing multi-drug resistant (MDR) Gram-negative ESKAPE bacteria in the faecal carriage and clinical samples from patients in an urban, tertiary and a rural, district hospital in uMgungundlovu District, KwaZulu-Natal, South Africa.

**Methods:**

This study took place in a district and tertiary hospital during a two-months period from May to June 2017 in uMgungundlovu district, South Africa. Rectal swabs collected from hospitalized patients, at admission, after 48 h and at discharge (whenever possible) formed the carriage sample while clinical isolates routinely processed in the microbiological laboratory during the sampling period were also collected and formed the clinical sample. Gram-negative ESKAPE bacteria were screened for ESBL production on selective MacConkey agar and confirmed using ROSCO kits. Minimum inhibitory concentrations were determined, and real-time and multiplex polymerase chain reaction were used to ascertain the presence of *bla*_CTX-M_ group-1-2-9, *bla*_CTX-M_ group 8/25, *bla*_SHV_, *bla*_TEM_, *bla*_OXA-1-like_, *bla*_KPC_, *bla*_VIM_, *bla*_IMP_, *bla*_GES_ and AmpC genes. Genomic fingerprinting was also performed using ERIC-PCR. Risk factors for ESBL-mediating MDR Gram-negative ESKAPE colonization were ascertained by univariate and multivariate logistic regression analyses.

**Results:**

Overall prevalence of carriage of ESBL-mediating MDR Gram-negative ESKAPE was 37.21% (16/43), 42.31% (11/26) and 57.14% (4/7) at admission, after 48 h and at discharge respectively. The prevalence of ESBL-mediating MDR Gram-negative ESKAPE bacteria in faecal carriage (46%) was higher than clinical samples (28%). Colonization was mainly associated with the referral from district to tertiary hospital with high statistical significance (OR: 14.40, 95% CI 0.98–210.84). *bla*_CTX-M-group-9_, *bla*_CTX-M-group-1_ and *bla*_SHV_ were the main resistance genes identified. Several patients carried more than two different isolates. A *Klebsiella pneumoniae* (K1) clone was circulating within wards and between hospitals.

**Conclusion:**

The study highlights the high prevalence of ESBL-mediating MDR Gram-negative ESKAPE bacteria in carriage and clinical samples among hospitalized patients in uMgungundlovu, South Africa. The wide dissemination of these resistant ESKAPE bacteria in hospitals necessitates improvements in routine screening and reinforcement of infection, prevention and control measures.

**Electronic supplementary material:**

The online version of this article (10.1186/s13756-018-0423-0) contains supplementary material, which is available to authorized users.

## Introduction

The selective pressure exerted using antibiotics and aggravated by the dearth of new active substances in the current therapeutic pipeline has led to a considerable increase in antibiotic resistance (ABR) worldwide [1, 2]. A small group of bacteria, i.e., *Enterococcus spp.*, *Staphylococcus aureus*, *Klebsiella pneumoniae*, *Acinetobacter baumannii*, *Pseudomonas aeruginosa* and *Enterobacter spp*., termed “ESKAPE” due to their ability to escape the activity of and develop high levels of resistance to multiple antibiotics, have recently gained global attention [3–5]. Of the six infamous ESKAPE pathogens, the four Gram-negative bacteria, i.e., *K. pneumoniae*, *A. baumannii*, *P. aeruginosa*, and *Enterobacter spp*., have been associated with four major types of multi-drug resistance (MDR), namely extended-spectrum β-lactamase (ESBL)-producing *K. pneumoniae* and *Enterobacter spp.*, carbapenemase-producing *A. baumannii* and metallo-β-lactamase producing *P. aeruginosa* (MBL-PA) which limit therapeutic options and negatively affect clinical outcomes [3–6]. Several resistance genes have been associated with the emergence of ESBL-mediating MDR Gram-negative ESKAPE bacteria globally. The bacterial production of enzyme hydrolysing antibiotics, particularly β-lactam antibiotics, is the most common mechanism of resistance in Gram-negative ESKAPE. Beta-lactamase enzymes have emerged following chromosomal mutation and acquisition of resistance genes carried on diverse mobile genetic elements (MGEs) such as plasmids, integrons, insertion sequences, transposons, genomic islands and bacteriophages [7]. The common transferability of resistance amongst bacteria will likely be associated with increasing rates of MDR infections and carriage, although some gaps remain as to the dissemination of multi-drug resistant bacteria in the community and among hospitalized patients.

MDR is increasingly being detected in numerous Gram-negative bacteria because of the extensive antibiotic use in communities and hospitals. Despite considerable efforts for their containment, ESBL-mediating multi-drug resistant Gram-negative ESKAPE bacteria are increasingly implicated in several difficult-to-treat infections in both developed and developing countries [5, 6, 8, 9] and were recently listed by the World Health Organization (WHO) as resistant bacteria of critical priority in research [3, 7, 8, 10]. Hospitals remain the main reservoir while immune-compromised patients such as those suffering from diabetes, chronic lung, kidney and cardiovascular diseases and cancers are the most affected. Whilst a better understanding on the impact of faecal carriage of ESBL-mediated resistance to Gram-negative ESKAPE bacteria is required, carriage is recognized as a potential risk for transmission and on subsequent development of infections especially in healthcare settings in developing countries due to inadequate infection, prevention and control measures.

In the African continent, antimicrobial resistance issue in general and MDR particularly, has not been adequately illustrated yet due limited financial resources. Knowledge of the burden of multidrug-resistant bacteria in South Africa could thus be valuable both to raise awareness on the necessity to prevent the spread of resistant infections in communities and hospitals, and to ameliorate empirical antibiotic therapy and clinical practice. This study seeks to compare the prevalence of faecal carriage of ESBL-mediated MDR Gram-negative ESKAPE bacteria among patients hospitalized in an urban, tertiary and a rural, district hospital in uMgungundlovu District, South Africa. In addition, the study is an attempt to provide insight into the risk factors associated with this carriage. Finally, the study assesses the phenotypic and genotypic characteristics and clonal relatedness of carriage and clinical ESBL-mediated MDR Gram-negative ESKAPE bacteria.

## Materials and methods

### Study population and settings

This study was conducted in a rural, district and urban, tertiary hospital, encoded for ethical reasons as H1 and H2, respectively, during 2 months from May 2017 to June 2017 in uMgungundlovu district, South Africa. The district hospital (H1) represents the smallest level of hospital and provides four services including obstetrics and gynaecology, paediatrics and child health, general surgery and general medicine with 141 beds. In contrast, the tertiary hospital (H2) offers several specialties, receives referral patients according to a nationally agreed referral plan and has approximately 505 beds.

### Patient enrolment and questionnaire survey

Total sampling was performed for the recruitment of participants i.e. all patients older than 18 years old, hospitalized in medical or surgical ward of the hospitals H1 and H2, and willing to participate were included in the study. Oral and written informed consent was obtained from all study participants after explanation of the procedure and purpose of the study. Patient information was gleaned from questionnaires completed by patients and data from patient records. Information was codified prior to analysis to maintain confidentiality.

### Sample collection

Sample collection took place in both surgical and general medical wards during a two-month period, 1 month at each of the hospitals. Rectal swabs that were collected aseptically with Amies swabs from symptomatic in-patients, at three-time points, at admission, after 48 h and at discharge (whenever possible) formed the carriage sample. Isolates from symptomatic patients originating from tissue, blood, urine, intravenous catheters, and sputum routinely processed in the microbiological laboratory during the sampling period formed the clinical sample.

### Definitions of terms

The specimen (blood, urine, sputum, tissue, intravenous catheter tips, fluid/aspirate and superficial swab) collected for diagnostic purpose from a symptomatic hospitalized patient was considered clinical sample. The clinical isolates were recovered from clinical samples obtained from patients hospitalized in various units of the selected hospitals. In contrast, carriage sample was the rectal swab collected from hospitalized patients at different time-points (admission, after 48 h and at discharge) out of diagnostic tests performed at hospitals.

### Laboratory analysis

#### Identification of gram-negative ESKAPE bacteria

During the sample collection, all rectal swabs were cultured onto MacConkey agar with and without cefotaxime (2 mg/L). After incubation for 18-24 h at 37 °C, each morphotype growing on MacConkey with cefotaxime (MCA + CTX) was subjected to Gram staining, catalase and oxidase tests, followed by biochemical identification with API 20E (bioMérieux, Marcy l’Etoile, France) and Vitek® 2 System (bioMérieux, Marcy l’Etoile, France) using the GN card according to the manufacturer’s instructions. Pure colonies were stored into Tryptone Soya Broth supplemented with 30% glycerol at − 20 °C for future use.

#### Phenotypic screening

All growing colonies were phenotypically screened for ESBL, AmpC, KPC, MBL, and OXA-48 production using ROSCO DIAGNOSTICA (Taastrup, Denmark) using 0.5 McFarland on Mueller-Hinton agar according to the manufacturer’s instructions.

#### Antimicrobial susceptibility testing

Minimum inhibitory concentrations (MICs) were determined via broth microdilution for all presumptive ESBLs and/or AmpCs, and/or MBL producers. Ampicillin, cefoxitin, cefuroxime, cefotaxime, ceftazidime, meropenem, imipenem, ertapenem, amikacin, gentamicin, ciprofloxacin, tigecycline, tetracycline, doxycycline, nitrofurantoin, and colistin constituted the antibiotic panel for carriage isolates. The Vitek® 2 System and Vitek® 2 Gram-negative Susceptibility card (AST-N255) were used to determining the MICs of clinical isolates. The results of MIC tests were interpreted according to the European Committee on Antimicrobial Susceptibility Testing (EUCAST) breakpoints [11] and the MDR which is the resistance to three or more classes of antibiotics was also assessed. *Escherichia coli* ATCC 25922, *K. pneumoniae* ATCC 700603 and *K. pneumoniae* ATCC 51503 were used as controls.

### Genomic characterization

#### Genomic extraction

Genomic DNA of selected strains were extracted using GenElute Bacterial Genomic DNA Kit (Sigma-Aldrich, St. Louis, MO, USA) according to the manufacturer’s instructions. Genomic DNA was stored at − 20 °C for future use.

#### Multiplex polymerase chain reaction (M-PCR)

The isolates were subjected to molecular testing using conventional and M-PCR assays to identify *bla*_CTX-M_ group 8/25 (*bla*_CTX-M-gp8/25_), *bla*_SHV_, *bla*_TEM_, *bla*_OXA-1-like_, *bla*_OXA-48_, *bla*_KPC_, *bla*_VIM_, *bla*_IMP_ and *bla*_GES_ genes as previously described [12] (Additional file 1: Table S1).

#### Real-time polymerase chain reaction (RT-PCR)

RT-PCR was performed to ascertain *bla*_AmpC_, *bla*_CTX-M-group-1_ (*bla*_CTX-M-gp1_), *bla*_CTX-M-group-2_ (*bla*_CTX-M-gp2_) and *bla*_CTX-M-group-9_ (*bla*_CTX-M-gp9_) resistance genes. Results were analysed on a programmable automate QuantStudio5™ (Applied Biosystems, CA, USA) using the Taqman Universal Master Mix 2× (Applied Biosystems, CA, USA) and ready-made assays (Thermo Scientific, CA, USA). Thermal temperature running conditions were as follows: UNG activation at 50 °C for 2 min, initial denaturation at 95 °C for 10 min, 30 cycles of denaturation at 95 °C for 10 s, annealing/extension at 60 °C for 1 min and a final extension at 60 °C for 30 s. The results were interpreted with QuantStudio™ design and analysis software version 1.4 (Applied Biosystems, CA, USA).

#### Genomic fingerprinting

Enterobacterial Repetitive Intergenic Consensus-Polymerase Chain Reaction (ERIC-PCR) was used to establish the link of different strains within and between hospitals, wards, carriage and clinical samples as well as across sampling points. The primers ERIC1 5’ATGTAAGCTCCTGGGGATTCAC3’ and ERIC2 5’AAGTAAGTGACTGGGGTGAGCG3’ [13] were used and PCR reactions were carried out in a 10 μl volume containing 5 μl of Dream*Taq* Green Polymerase Master Mix 2X (Thermo Fisher Scientific, Johannesburg, South Africa), 2.8 μl of nuclease free water, 0.1 μl of each primer (100 μM), and 2 μl of DNA template. The reactions were carried out with the following cycling conditions: initial denaturation at 94 °C for 3 min, 30 cycles consisting of a denaturation step at 94 °C for 30 s, annealing at 50 °C for 1 min, extension at 65 °C for 8 min, a final extension step at 65 °C for 16 min and final storage at 4 °C. The generated amplicons were resolved by horizontal electrophoresis on 1.5% (wt/vol) Tris-Borate-EDTA (Merck, Germany) agarose gels together with the Quick-load®1-kb (Biolabs, New England) and run in an electric field of 110 V for 2 h 30 min. Electrophoresis gels were visualized by a UV light trans-illuminator, images were captured using a Gel Doc™ XR+ system (BioRad Laboratories, CA, Foster City, USA) and analysed by Image Lab™ Software (version 4.0, BioRad Laboratories, CA, Foster City, USA).

ERIC-PCR profiles were normalized using the Quick-load®1-kb (Biolabs, New England) DNA molecular weight marker as the external standard. For cluster analysis, data were exported to Bionumerics software (version 7.6, Applied Maths, TX, USA). Strains were allocated to different clusters by calculating the similarity coefficient from the homology matrix using the Jaccard method. Dendrograms were constructed based on the average linkages of the matrix and using the Unweighted Pair-Group Method (UPGMA). Optimization and band tolerance were set at 1% (version 7.6, Applied Maths, TX, USA) and 80% similarity cut-off was used to define clusters.

### Data analysis

Data was coded and entered on an Excel spreadsheet (Microsoft Office 2016) and analysed using STATA (version 14.0, STATA Corporation, TX, USA). Risk factors for ESBL-mediating MDR Gram-negative ESKAPE colonization were ascertained by univariate and multivariate logistic regression analyses. Prevalence of MDR carriage was compared between categories (viz. hospital, ward and time-point) using the chi-square and Fisher’s exact test as appropriate. A *p*-value < 0.05 was regarded as statistically significant.

## Results

### Population characteristics

A total of 75 hospitalized patients were contacted, amongst whom, 45 (60%) agreed to participate, answered the questionnaire and provided samples. Out of the 45 patients enrolled, faecal carriage was collected from 21 female and 24 were males, and the district hospital (*n* = 27) accounted more participant than the tertiary hospital (*n* = 18). The main reasons of hospitalization were cancer, cellulitis, hypoglycaemia, renal failure, diabetes, breath disorder, surgery and wound, in both hospitals. The patient’s follow-up rate was 96%, 58% and 16% of rectal swabs collected at admission, after 48 h and at discharge, respectively.

The overall prevalence of carriage was 37.21% (16/43), 42.31% (11/26) and 57.14% (4/7) at admission, after 48 h and at discharge, respectively, with males being more colonized than females as were patients referred from another hospital (Table 1). Patients in the tertiary hospital were more likely to be colonized by MDR ESKAPE bacteria at admission (50%) and discharge (66.66%) than those of the district hospital (Table 1). Furthermore, patients admitted to the general medical ward were more colonized in the district hospital at all time-points whereas, in the tertiary hospital, the prevalence in the surgical ward was higher at admission and discharge. In parallel, the prevalence of MDR ESKAPE bacteria in faecal carriage (46%) was higher than clinical samples (28%).

**Table 1:** Fecal carriage of resistant Gram-negative ESKAPE bacteria isolated from hospitalized patients in a rural district and a tertiary urban hospital

Variables	District Rural Hospital *n* = 27						Tertiary Urban Hospital *n* = 18					
	Admission, (%)	*p*	After 48 h, (%)	*p*	At discharge, (%)	P	Admission, (%)	*p*	After 48 h, (%)	*p*	At discharge, (%)	*P*
Overall	29.63	….	47.05	…..	50	….	50	…	33.33	…	66.66	…
Socio-demographic factors												
*Gender*												
Female	21.4	0.333	33	0.229	50	….	40	0.590	50	0.571	50	0.386
Male	38.4		63		0		55		29		100	
Clinical history												
*Previous hospitalization (within one year)*												
Yes	13	0.206	0	0.012	0	0.248	20	0.106	25	0.635	100	0.386
No	37		67		66.67		64		40		50	
*Antibiotic use (during hospital stay)*												
Yes	27.78	0.766	33.33	0.402	50	1.000	33	0.522	33	1.000	100	0.386
No	33.33		54.55		50		54		33		50	
*Transferred from another hospital*												
Yes	100	0.116	0	…	0	….	75	0.046	50	0.343	100	0.386
No	27		47		50		25		20		50	
*Hospital ward*												
Medicine	40	0.187	56	0.457	50	1.000	40	0.590	40	0.635	50	0.386
Surgery	17		38		50		55		25		100	

Out of the 45 patients enrolled, some refused rectal sampling, some were discharged or transferred after 48 h, while other could not be sampled due to their condition, leading to variability in number

### Risk factors for MDR gram-negative ESKAPE bacteria carriage

Patients referred from the district to the tertiary hospital had an increased the risk of being colonized by resistant bacteria at admission (OR = 9, 95% CI 4.68–17.30) and after 48 h (OR = 4; 95% CI 1.50–10.66, Table 2). Similarly, the gender (male) increases the odds of being colonized at admission and after 48 h in district hospital (Table 2).

**Table 2:** Risk factors associated with faecal carriage of ESBL-producing Gram-negative ESKAPE bacteria (Univariate Logistic regression)

Variables	District hospital		Tertiary hospital	
	Admission	After 48 h	Admission	After 48 h
	OR (95% CI)	OR (95% CI)	OR (95% CI)	OR (95% CI)
Gender (F or M)	2.29 (0.42–12.50)	3.33 (0.45–24.44)	1.8 (0.21–15.40)	0.4 (0.16–10.02)
Antibiotic use (Yes or No)	1.3 (0.23–7.32)	0.42 (0.05–3.31)	0.43 (0.03–5.98)	1
Co-morbidity	1.05 (0.61–1.83)	1.03 (0.48–2.24)	1.05 (0.61–1.83)	1.03 (0.48–2.24)
Previous hospitalization	0.24 (0.02–2.40)	1	0.14 (0.01–1.76)	0.5 (0.03–8.95)
Transferred from another hospital	1	1	9 (0.93–86.52)	4 (0.21–75.67)
Ward (Medicine or Surgery)	0.3 (0.05–1.88)	0.48 (0.07–3.35)	1.8 (0.21–15.40)	0.5 (0.03–8.95)

The multivariate analysis further confirmed that referral from district to tertiary hospital was significantly associated with MDR Gram-negative ESKAPE bacteria at admission (OR = 14.40, 95% CI 1. 0.98–210.84) and after 48 h (OR = 5.72, 95% CI 0.17–189.00) as was the gender for these two time-points in the district hospital (Table 3).

**Table 3:** Predictive risk factors associated with fecal carriage of MDR Gram-negative ESKAPE bacteria in a district and tertiary hospital (Multivariate Logistic regression)

Variables	District Hospital		Tertiary Hospital	
	Admission; OR (95% CI)	After 48 h; OR (95% CI)	Admission; OR (95% CI)	After 48 h; OR (95% CI)
Gender (F or M)	7.12 (0.54–93.75)	3.61 (0.34–37.83)	1.21 (0.09–15.61)	0.29 (0.005–16.27)
Antibiotic use (Yes or No)	4.73 (0.28–80.57)	0.93 (0.08–11.40)	0.26 (0.007–9.01)	0.41 (0.009–17.46)
Transferred from another hospital	1	1	14.40 (0.98–210.84)	5.72 (0.17–189.00)
Hospital Ward (Medicine or Surgery)	0.08 (0.004–1.39)	0.42 (0.05–3.81)	2.09 (0.10–42.29)	1.14 (0.03–49.14)

### Prevalence of MDR gram-negative ESKAPE bacteria

Out of 159 non-duplicates resistant Gram-negative bacteria isolated, 31 (19.50%) were MDR Gram-negative ESKAPE bacteria of which 21 (67.74%) were clinical isolates (11 tissue, 2 bloods, 3 urines, 3 intravenous catheters, 2 sputum) obtained after 48 h from hospitalized patients (15 males and 6 females) with symptomatic infections in different departments (medicine, surgery, intensive care units). Ten (32.26%) MDR Gram-negative ESKAPE bacteria were isolated from the rectal swab of in-patients (6 females, 4 males). In the district hospital, seven isolates were identified, five (71.43%) in carriage and two (28.57%) in clinical samples. *K. pneumoniae* (*n* = 2) and *E. aerogenes* (*n* = 2) were the main bacterial species isolated in carriage samples while *E. cloacae* (*n* = 2) was the sole clinical isolates*.* In contrast, in the tertiary hospital, five (20.83%) isolates were identified in carriage and 19 (79.16%) in clinical samples. The main pathogen identified in carriage was *E. aerogenes* (*n* = 2) while *P. aeruginosa* (*n* = 7) and *A. baumannii* (*n* = 7) were the main clinical isolates.

### Antimicrobial resistance profiles

In the tertiary hospital, especially in the medical ward, isolates expressed high resistance to ampicillin (100%), cefuroxime (100%) and cefotaxime (100%) in both carriage and clinical samples (Table 4). Similarly, in the surgical ward of the same hospital, clinical samples showed high resistance to ampicillin (100%), cefuroxime (100%), cefotaxime (88%), cefoxitin (88%), and nitrofurantoin (55%) while the unique carriage isolate was resistant to all the panel of antibiotics tested.

**Table 4:** Resistance to selected antibiotics in ESBL-producing Gram-negative ESKAPE bacteria isolated from carriage and clinical samples in a district and tertiary hospital

Antibiotics	Tertiary hospital								District hospital					
	Medical ward				Surgical ward				Medical ward				Surgical ward	
	Carriage		Clinical		Carriage		Clinical		Carriage		Clinical		Carriage	
	MIC (μg/ml) range	No. resistant isolates (%)	MIC (μg/ml) range	No. resistant isolates (%)	MIC (μg/ml) range	No. resistant isolates (%)	MIC (μg/ml) range	No. resistant isolates (%)	MIC (μg/ml) range	No. resistant isolates (%)	MIC (μg/ml) range	No. resistant isolates (%)	MIC (μg/ml) range	No. resistant isolates (%)
Ampicillin	≥512	4 (100)	≥32	11 (100)	≥512	1 (100)	16–32	8 (100)	≥512	4 (100)	16- ≥ 32	2 (100)	≥512	1 (100)
Cefoxitin	8–512	3 (75)	4	7 (64)	16	1 (100)	8- ≥ 64	7 (88)	128 ≥ 512	4 (100)	≥64	2 (100)	≥512	1 (100)
Cefuroxime	≥512	4 (100)	≥64	11 (100)	128	1 (100)	8–64	8 (100)	256 ≥ 512	4 (100)	16- ≥ 64	2 (100)	≥512	1 (100)
Cefotaxime	≥512	4 (100)	≥64	11 (100)	128	1 (100)	1- ≥ 64	7 (88)	32- ≥ 512	4 (100)	< 1–32	2 (100)	≥512	1 (100)
Ceftazidime	512	4 (100)	≥64	8 (73)	256	1 (100)	1- ≥ 64	4 (50)	32- ≥ 512	4 (100)	≤1–16	2 (100)	≥512	1 (100)
Meropenem	0.5–2	2(50)	0.25	3 (27)	2	1 (100)	0.25 ≥ 16	4 (50)	0.25–16	2 (50)	0.25	0 (0)	16	1 (100)
Imipenem	4–8	4 (100)	0.25	3 (27)	16	1 (100)	0.5- ≥ 16	3 (37.3)	2–32	2 (50)	0.25–1	0 (0)	64	1 (100)
Ertapenem	1–2	4 (100)	0.5	0 (0)	16	1 (100)	≤0.5	0 (0)	0.25–8	3 (75)	0.5	0 (0)	64	1 (100)
Amikacin	8–128	4 (100)	8	2 (18)	64	1 (100)	2- ≥ 64	4 (50)	8–128	2 (100)	2	0 (0)	≥512	1 (100)
Gentamicin	128	4 (100)	≥16	8 (73)	8	1 (100)	1- ≥ 16	4 (50)	4–16	4 (100)	1	0 (0)	≥512	1 (100)
Ciprofloxacin	64–512	4 (100)	≥4	6 (55)	32	1 (100)	0.25- ≥ 4	5 (45)	0.5–64	3 (75)	≤0.25	0 (0)	32	1 (100)
Tigecycline	16–64	4 (100)	1	4 (36)	16	1 (100)	0.5- ≥ 8	3 (37.3)	2–64	4 (100)	1	0 (0)	8	1 (100)
Nitrofurantoin	≥512	4 (100)	128	9 (82)	≥512	1 (100)	≥512	6 (55)	≥512	4 (100)	16- ≥ 512	0 (0)	≥512	1 (100)
Colistin	8–512	4 (100)	0.5	0 (0)	8	1 (100)	≤0.5	0(0)	0(0)	0(0)	≤0.5	0 (0)	≤0.5	0

In the district hospital, the isolate identified in carriage samples in the surgical ward displayed maximum resistance (100%) to all antibiotics except colistin while those detected in medical ward exhibited high level of resistance to ampicillin (100%), cefuroxime (100%), cefotaxime (100%), ceftazidime (100%), cefoxitin (100%), amikacin (100%), gentamicin (100%), nitrofurantoin (100%) and tigecycline (100%) (Table 4).

### Genetic diversity of isolated MDR strains

**Fig. 1:**
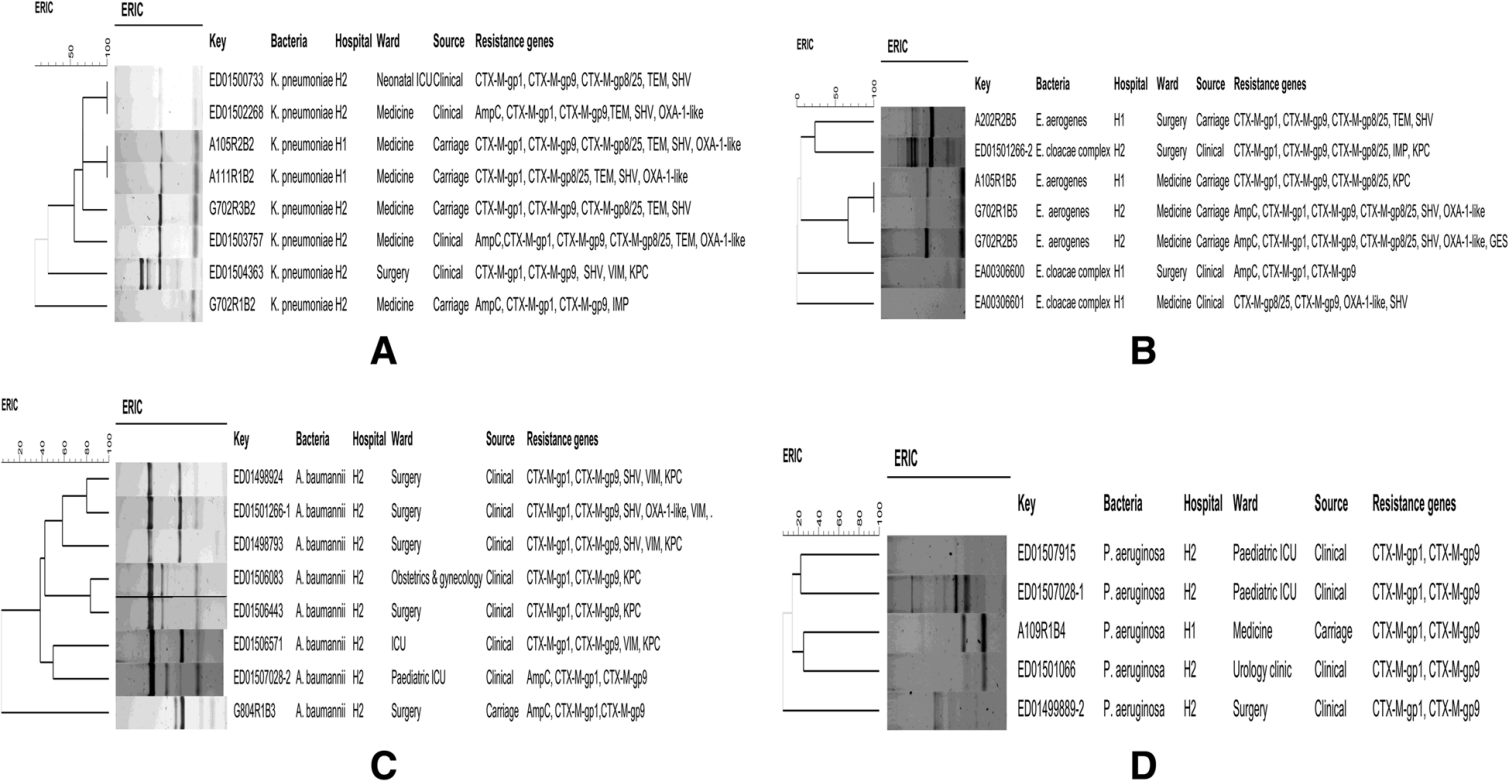
Dendrograms of ESBL-producing of Gram-negative ESKAPE bacteria isolated from carriage and clinical samples from hospitalized patients. **a** *K. pneumoniae*, (**b**) *E. aerogenes* and *cloacae*, (**c**) *A. baumannii*, (**d)** *P. aeruginosa*

Overall, the predominant ESBL genes were *bla*_CTX-M-gp9_ (90%, 28/31), *bla*_CTX-M-gp1_ (71%, 22/31), *bla*_SHV_ (42%, 13/31), *bla*_CTX-M-gp8/25_ (36%, 11/31), *bla*_OXA-1-Like_ (29%, 9/31) and *bla*_TEM_ (23%, 7/31) for both carriage and clinical samples. In the tertiary hospital, *bla*_CTX-M-gp9_ (100%), *bla*_CTX-M-gp1_ (87.5%), *bla*_KPC_ (75%) and *bla*_VIM_ (50%) were the main resistance genes detected in *A. baumannii* while *K. pneumoniae* strains harboured mainly *bla*_CTX-M-gp8/25_ (66.6%), *bla*_TEM_ (66.6%), *bla*_SHV_ (66.6%), *bla*_CTX-M-gp9_ (50%) and *bla*_CTX-M-gp1_ (50%) (Table 5). It is noteworthy to mention that all isolates harboured at least two resistance genes and a maximum of seven genes were detected in one *E. aerogenes* (G702R2B5) isolate (Fig. 1b). In the district hospital, *bla*_CTX-M-gp9_ (100%), *bla*_SHV_ (100%), and *bla*_TEM_ (100%) were the predominant genes in *K. pneumoniae* whereas *bla*_CTX-M-gp9_ (100%), *bla*_OXA-1-Like_ (50%), *bla*_CTX-M-gp1_ (50%) and *bla*_CTX-M-gp8/25_ (50%) were the main genes identified in *E. cloacae* (Table 5).

**Table 5:** Resistance genes in ESBL-producing Gram-negative ESKAPE bacteria

Bacteria	No. of strains, *n* = 31 (%)	Resistance genes, *n* (%)									
		AmpC	TEM	SHV	CTX-M group-1	CTX-M group-9	CTX-M Group 8/25	IMP	VIM	KPC	OXA-1-like
Tertiary hospital (*n* = 24)											
*K. pneumoniae*	6 (25)	2 (33.3)	4 (66.6)	4 (66.6)	3 (50)	3 (50)	4 (66.6)	1 (16.6)	1 (16.6)	1 (16.6)	2 (33.33)
*A. baumannii*	8 (33.3)	3 (37.5)	/	3 (37.5)	7 (87.5)	8 (100)	/	/	4 (50)	6 (75)	2 (25)
*P. aeruginosa*	7 (29.5)	1 (14.28)	/	/	7 (100)	7 (100)	/	/	/	/	/
*E. aerogenes*	2 (8.33)	2 (100)	/	2 (100)	/	2 (100)	2 (100)	/	/	/	2 (100)
*E. cloacae*	1 (4.16)	/	/	/	1 (100)	1 (100)	1 (100)	1 (100)	/	1 (100)	/
District hospital (n = 7)											
*K. pneumoniae*	2 (28.57)	/	2 (100)	2 (100)	1 (50)	2 (100)	1 (50)	/	/	/	2 (33.33)
*P. aeruginosa*	1 (14.28)	/	/	/	1 (100)	1 (100)	/	/	/	/	/
*E. aerogenes*	2 (28.57)	1 (50)	1 (50)	1 (50)	1 (50)	2 (100)	2 (100)	/	/	/	/
*E. cloacae*	2 (28.57)	1 (50)	/	1 (50)	1 (50)	2 (100)	1 (50)	/	/	/	1 (50)

### Genomic fingerprint

ERIC-profiles revealed some associations within species and suggest a likely transmission of resistant ESKAPE bacteria across patients, wards and hospitals (Additional file 2: Table S2). For *K. pneumoniae*, one main cluster showing high genetic similarities was observed (Fig. 1a). *K. pneumoniae* strains A111R1B2 and A105R2B2 detected among two patients at admission for the former and after 48 h for the latter, in the medical ward of the district hospital showed 100% of similarity and shared common ancestors with one carriage and three clinical strains isolated in the tertiary hospital (Fig. 1a and Additional file 2: Table S2). Similarly, one pair of *E. aerogenes*, A105R1B5 and G702R1B5 isolated from two patients in the medical ward of the district and tertiary hospital, both at admission, also exhibited 100% of similarity and shared a common ancestor with another strain G702R2B5 collected after 48 h (Fig. 1b). Although, *A. baumannii* (Fig. 1c) and *P. aeruginosa* (Fig. 1d) were more genetically diverse, some isolates shared a common ancestor within and between the carriage and clinical samples.

## Discussion

The overall prevalence of carriage at admission was 37.21% (16/43) and we found that 42.31% (11/26) and 57.14% (4/7) were still MDR ESKAPE carriers after 48 h and at discharge. Notwithstanding the small sample size, our results showed that the carriage of MDR Gram-negative ESKPAPE bacteria increased with the hospital length of stay. Our results are consistent with a Norwegian prospective cohort study carried out from 2009 to 2011 investigating the risk factors for and duration of prolonged faecal carriage of ESBL-producing *K. pneumoniae* amongst patients with community acquired urinary tract infections which revealed high prevalence of ESBL faecal carriage (ranging from 15 to 61%) at six different time points [14].

At hospital level, the rate of carriage at admission in the district hospital (30%) compared with the tertiary hospital (50%) suggests that patients admitted to the tertiary hospital are likely to be more colonized by MDR Gram-negative ESKAPE bacteria than those of the district healthcare facility (Table 1). Our findings could be explained by the fact that all patients admitted to this level of the hospital are generally transferred from lower level healthcare facilities of the South African health system. This is further confirmed by the increased odds of being colonized in the univariate and multivariate analysis. Similarly, at discharge, patients of the tertiary hospital (67%) were more colonized than those of the district hospital (50%). This could be explained by the complexity of cases associated with invasive medical procedures and greater antibiotic use in the tertiary hospital. However, after 48 h, the prevalence of carriage was higher in patients in the district hospital (47%) compared with the tertiary hospital (33%) intimating. This contrast could point out sub-optimal infection prevention and control measures in this level of healthcare setting. Besides, tertiary hospital with its more complicated cases and subsequent higher antibiotic use would have likely created greater selection pressure for resistance, but an anomalously greater resistance was observed in carriage samples in the district hospital. The small sample numbers preclude nonetheless definitive conclusions about carriage rates and resistance patterns.

The prevalence of MDR Gram-negative ESKAPE bacteria in faecal carriage (46%) was higher than that of clinical samples (28%) during the study period. Faecal carriage of resistant bacteria has been demonstrated to precede infections and consequently, such high prevalence of asymptomatic faecal carriage is of critical significance. Our results concur with a study from France where the prevalence of MDR Gram-negative bacilli isolated from stool samples was higher than that of clinical samples during a non-outbreak situation in a French Hospital [15]. They are however higher than a report from Mahomed and Coovadia (2014) which demonstrated 4.7% of faecal carriage of ESBL producing *Enterobacteriaceae* amongst children from the community in KwaZulu-Natal, South Africa [16]. Our findings may be an underestimation because of different diagnostic, stewardship practices, preference for empirical treatment and budget constraints such that not every infection generates a microbiological sample.

During the two-months period, 21 clinically relevant MDR Gram-negative ESKAPE bacteria out of 74 isolates were identified in both hospitals. Moreover, the prevalence of MDR *A. baumannii* and *P. aeruginosa* were 41.61% (10 out of 21 MDR Gram-negative ESKAPE bacteria) and 33.33% (7 out of 21 MDR Gram-negative ESKAPE bacteria) in clinical samples, respectively. The isolation of three *A. baumannii* strains, cluster A1, from tissue of three different patients (ED01498924, ED01498793, ED01498924) in surgery, consolidate the likely dissemination of this cluster within this ward in the tertiary hospital (Additional file 2: Table S2 and Fig. 1c).

In carriage samples, MDR *K. pneumoniae* and *Enterobacter spp.* were the predominant bacteria in both hospitals. This is consistent with a South African study where *K. pneumoniae* was the main pathogen identified in stool samples of children from the community of KwaZulu-Natal, South Africa [16]. Similarly, a 68% prevalence of ESBL-producing *Enterobacteriaceae* faecal carriage was shown amongst Egyptian patients with community-acquired gastrointestinal complaints [17].

An interesting finding was the inter-hospital and inter-patient spread of *K. pneumoniae* (cluster K1) in carriage, which were isolated from two patients (A105R2B2 and A111R1B2) hospitalized in general medicine in district hospital, sharing common ancestor with a patient (G702R3B2) from tertiary hospital (Additional file 2: Table S2). Interestingly, the isolated strains were identified in the medical ward and at different time-points, confirming the dissemination of this cluster across hospitals. In addition, *K. pneumoniae* strains from the same cluster (K1) were detected in urine (ED01500733) and sputum (ED01502268) of clinically ill patients hospitalized in intensive care unit (ICU) and medical ward in the tertiary hospital, respectively. This suggests that the *K. pneumoniae* K1 strains is circulating within wards and hospitals, and consequently could probably be source of nosocomial infections in hospitals.

Two patients, A105R1B5 and G702R1B5 also carried *Enterobacter spp.* (cluster E2) at admission in both district and tertiary hospitals, specifically in the medical wards (Additional file 2: Table S2) intimating the emergence of these strains in the community with subsequent entry into the district hospital, as the first level of care, and followed by spread to the tertiary hospital through referral. This result is consistent with our analyses which demonstrated that in the district hospital, the main risk factors were antibiotic use and gender while the referral and hospital ward were the principal risk factors at tertiary level (Tables 2 and 3).

Overall, the predominant ESBLs detected in carriage were *bla*_CTX-M-gp9_ (90%), *bla*_SHV_ (60%), *bla*_CTX-M-gp1_(50%), *bla*_TEM_ (40%) and *bla*_OXA-1-like_ (40%). CTX-M is predominantly reported in community-acquired infections which would be more prevalent in the district hospital as the first level of care. These results are consistent with global reports. For instance, *bla*_CTX-M-group_ were recently observed in adults in a community in Netherlands and ambulatory patients in Egypt with both gastrointestinal complaints [17, 18]. Similarly, studies from Guinee-Bissau, Niger, Gabon and Tanzania, reported high prevalence of ESBL faecal carriage with *bla*_CTX-M_, *bla*_TEM_ and *bla*_SHV_ being the main genes identified [19–21]. The prevalence of AmpC was also higher in carriage (40%) compared to clinical samples (23.80%). Finally, carbapenemases were identified in clinical samples for in these hospitals, specifically, KPC and VIM in clinical *A. baumannii* isolates as well as IMP in a carriage *K. pneumoniae* isolate. An *E. aerogenes* isolate further showed *bla*_GES_ along with *bla*_CTX-M-gp1_, *bla*_CTX-M-gp9_, *bla*_CTX-M-gp8/25_, *bla*_SHV_ and *bla*_OXA-1-like_ in a carriage sample. The faecal carriage of MDR Gram-negative ESKAPE bacteria appears to be a source of cross-transmission between patients. The substantial genetic similarity within and between carriage and clinical isolates as well as wards and hospital settings reveal their potential implications in future outbreak situations that may occur either in hospitals or in communities. Efforts should thus be made amongst communities and asymptomatic patients for better containment of antibiotic resistance dissemination.

Gender, antibiotic use, type of healthcare settings and referral from another hospital were the main risk factors identified. These results suggest that routine screening for MDR Gram-negative ESKAPE bacteria at admission should be implemented, and infection, prevention and control measures reinforced to prevent potential outbreaks by these resistant pathogens [22].

## Conclusion

This study highlights the high prevalence of ESBL-mediating MDR Gram-negative ESKAPE bacteria in carriage and clinical samples among hospitalized patients in uMgungundlovu. It is imperative to implement regular screening and surveillance of MDR Gram-negative ESKAPE bacteria in communities and hospitals, to monitor epidemiological changes, ascertain socio-economic impact and inform antibiotic treatment. These screening and surveillance measures coupled with strict infection prevention and control programmes and antimicrobial stewardship programmes (ASP) are essential to address antibiotic resistance in these settings.

## Additional files

Additional file 1: **Table S1.** Oligonucleotide sequences for ESBL and carbapenemase resistance genes included in multiplex PCR assays. (DOCX 17 kb)

Additional file 2: **Table S2.** Antibiotic Resistance Profiles and Resistance Genes of Isolates from Single Patients. (DOCX 38 kb)
