# Supplementary material for: Extended spectrum beta-lactamase mediated resistance in carriage and clinical gram-negative ESKAPE bacteria: a comparative study between a district and tertiary hospital in South Africa
Source: Antimicrob Resist Infect Control. 2018 Nov 14;7:134. doi: 10.1186/s13756-018-0423-0 (PMC6237030; doi:10.1186/s13756-018-0423-0)
Supplement: Supplementary file 1 — Table S1. Oligonucleotide sequences for ESBL and carbapenemase resistance genes included in multiplex PCR assays. (DOCX 17 kb) [file 13756_2018_423_MOESM1_ESM.docx]

| PCR name | Target gene | Primer name | Sequence (5’-3’) | Amplicon size (bp) | Ref. |
| --- | --- | --- | --- | --- | --- |
| Multiplex I  TEM, SHV,  OXA-1-like | TEM -1 and TEM-2 | MultiTSO-T_for  MultiTSO-T_rev | CATTTCCGTGTCGCCCTTATTC  CGTTCATCCATAGTTGCCTGAC | 800 | 13 |
|  | SHV-1 | MultiTSO-S_for  MultiTSO-S_rev | AGCCGCTTGAGCAAATTAAAC  ATCCCGCAGATAAATCACCAC | 713 |  |
|  | OXA-1, OXA-4 and OXA-40 | MultiTSO-O_for  MultiTSO-O_rev | GGCACCAGATTCAACTTTCAAG  GACCCCAAGTTTCCTGTAAGTG | 564 |  |
| Multiplex II  CTX-M group 8/25 | CTX-M-8, CTX-M-25, CTX-M-26 and  CTX-M-39 to CTX-M-41 | CTX-Mg8/25_for  CTX-Mg8/25_rev | AACRCRCAGACGCTCTAC^a^  TCGAGCCGGAASGTGTYAT^a^ | 326 |  |
| Multiplex III  GES and  OXA-48-like | GES-1 to GES-9 and GES-11 | MultiGES_for  MultiGES_rev | AGTCGGCTAGACCGGAAAG  TTTGTCCGTGCTCAGGAT | 399 |  |
|  | OXA-48-like | MultiOXA-48_for  MultiOXA-48_rev | GCTTGATCGCCCTCGATT  GATTTGCTCCGTGGCCGAAA | 281 |  |
| Multiplex IV  IMP, VIM and KPC | IMP variants except  IMP-9, IMP-16, IMP-18, IMP-22 and IMP-25 | MultiIMP_for  MultiIMP_rev | TTGACACTCCATTTACDG^a^  GATYGAGAATTAAGCCACYCT^a^ | 139 |  |
|  | VIM variants including VIM-1 and VIM-2 | MultiVIM_for  MultiVIM_revc | GATGGTGTTTGGTCGCATA  CGAATGCGCAGCACCAG | 390 |  |
|  | KPC-1 to KPC-5 | MultiKPC_for  MultiKPC_rev | CATTCAAGGGCTTTCTTGCTGC  ACGACGGCATAGTCATTTGC | 538 |  |

Additional file 1 **Table S1** Oligonucleotide sequences for ESBL and carbapenemase resistance genes included in multiplex PCR assays

^a^Y=T or C; R=A or G; S=G or C; D=A or G or T.
