# Supplementary material for: Extended spectrum beta-lactamase mediated resistance in carriage and clinical gram-negative ESKAPE bacteria: a comparative study between a district and tertiary hospital in South Africa
Source: Antimicrob Resist Infect Control. 2018 Nov 14;7:134. doi: 10.1186/s13756-018-0423-0 (PMC6237030; doi:10.1186/s13756-018-0423-0)
Supplement: Supplementary file 2 — Table S2. Antibiotic Resistance Profiles and Resistance Genes of Isolates from Single Patients. (DOCX 38 kb) [file 13756_2018_423_MOESM2_ESM.docx]

**Additional file 2: Table S2. Antibiotic Resistance Profiles and Resistance Genes of Isolates from Single Patients**

| **Patient ID** | **Isolate (ID code)** | **Hospital** | **Ward** | **Source** | **MIC values (µg/ml)** | | | | | | | | | | **Beta-lactamase resistance genes** | | | | | | | | | **ERIC cluster** | |
| --- | --- | --- | --- | --- | --- | --- | --- | --- | --- | --- | --- | --- | --- | --- | --- | --- | --- | --- | --- | --- | --- | --- | --- | --- | --- |
|  |  |  |  |  | **Ampicillin** | **Cefoxitin** | **Cefotaxime** | **Ceftazidime** | **Imipenem** | **Meropenem** | **Gentamicin** | **Amikacin** | **Ciprofloxacin** | **Tigecycline** | **AmpC** | **TEM** | **SHV** | **CTX-M gp1** | **CTX-M gp9** | **CTX-M gp8/25** | **OXA-1-like** | **Carbapenemases** |  | |  |
| **Carriage samples** | | | | | | | | | | | | | | | | | | | | | | | | | |
| A105 | *E. aerogenes* (A105R1B5) | District | Medicine | Admission | ≥512 | ≥512 | ≥512 | ≥512 | 16 | 16 | 16 | 32 | 64 | 16 | **-** | **-** | **-** | **+** | **+** | **+** | **+** | KPC | **E2** | |  |
|  | *K. pneumoniae* (A105R2B2) |  |  | After 48h | ≥512 | ≥512 | ≥512 | ≥512 | 32 | 16 | 8 | 8 | 64 | 64 | **-** | **+** | **+** | **+** | **+** | **+** | **+** | - | **K1** | |  |
| A109 | *P. aeruginosa* (A109R1B4) | District | Medicine | Admission | ≥512 | 128 | 32 | 32 | 2 | 1 | 8 | 128 | 0.5 | 2 | **-** | **-** | **-** | **+** | **+** | **-** | **-** | - | P2 | |  |
| A111 | *K. pneumoniae* (A111R1B2) | District | Medicine | Admission | ≥512 | ≥512 | ≥512 | 32 | 2 | 0.25 | 4 | 8 | 32 | 16 | **-** | **+** | **+** | **+** | **-** | **+** | **+** | - | **K1** | |  |
| A202 | *E. aerogenes* (A202R2B5) | District | Surgery | After 48h | ≥512 | ≥512 | ≥512 | ≥512 | 64 | 16 | ≥512 | ≥512 | 32 | 8 | **-** | **+** | **+** | **+** | **+** | **+** | **-** | - | **E1** | |  |
| G702 | *K. pneumoniae* (G702R1B2) | Tertiary | Medicine | Admission | ≥512 | ≥512 | ≥512 | ≥512 | 4 | 2 | ≥512 | 128 | ≥512 | 16 | **+** | **-** | **-** | **+** | **+** | **-** | **-** | IMP | **K3** | |  |
|  | *E. aerogenes* (G702R1B5) |  |  | Admission | ≥512 | 64 | ≥512 | ≥512 | 8 | 0.5 | 128 | 32 | ≥512 | 32 | **+** | **-** | **+** | **+** | **+** | **+** | **+** | - | **E2** | |  |
|  | *E. aerogenes* (G702R2B5) |  |  | After 48h | ≥512 | ≥512 | ≥512 | ≥512 | 4 | 2 | ≥512 | 128 | ≥512 | 16 | **+** | **-** | **-** | **+** | **+** | **+** | **+** | GES | **E2** | |  |
|  | *K. pneumoniae* (G702R3B2) |  |  | Discharge | ≥512 | 8 | ≥512 | 512 | 64 | 0.5 | 512 | 8 | 64 | 64 | **-** | **+** | **+** | **+** | **+** | **+** | **+** | - | **K1** | |  |
| G804 | *A. baumannii* (G804R1B3) | Tertiary | Surgery | Admission | ≥512 | 16 | 128 | 256 | 16 | 2 | 8 | 64 | 32 | 16 | **+** | **-** | **-** | **+** | **+** | **-** | **-** | - | A3 | |  |
| **Clinical samples** | | | | | | | | | | | | | | | | | | | | | | | | | |
| ED01498924 | *A. baumannii* | Tertiary | Surgery | Tissue | ≥32 | ≥64 | ≥64 | ≥64 | ≥16 | ≥16 | ≥16 | ≥64 | ≥4 | ≤0.5 | **-** | **-** | **+** | **+** | **+** | **-** | **-** | VIM, KPC | **A1** | |  |
| EA00306601 | *E. cloacae* | District | Medicine | Blood | ≥32 | ≥64 | 32 | 16 | 1 | ≤0.25 | ≤1 | ≤2 | ≤0.25 | 1 | **-** | **-** | **+** | **-** | **+** | **+** | **+** | - | E4 | |  |
| EA00306600 | *E. cloacae* | District | Medicine | Blood | ≥32 | ≥64 | 32 | 16 | 0.5 | ≤0.25 | ≤0.25 | ≤2 | ≤0.25 | 1 | **+** | **-** | **-** | **+** | **+** | **-** | **-** | - | E3 | |  |
| ED01499889-2 | *P. aeruginosa* | Tertiary | Surgery | Suppuration | ≥32 | 32 | 32 | 2 | 0.5 | ≤0.25 | 2 | 8 | ≥4 | 4 | **-** | **-** | **-** | **+** | **+** | **-** | **-** | - | P3 | |  |
| ED01501266-2 | *E. cloacae* | Tertiary | Surgery | Suppuration | 16 | ≥64 | ≤1 | ≤1 | 0.5 | ≥16 | ≤2 | ≤1 | ≤0.25 | 1 | **-** | **-** | **-** | **+** | **+** | **+** | **-** | - | **E1** | |  |
| ED01500733 | *K. pneumoniae* | Tertiary | ICU | Urine | ≥32 | ≤4 | ≥64 | 16 | ≤0.25 | ≤0.25 | ≥16 | ≤2 | 2 | ≤0.5 | **-** | **+** | **+** | **+** | **+** | **+** | **-** | - | **K1** | |  |
| ED01498793 | *A. baumannii* | Tertiary | Surgery | Tissue | ≥32 | ≥64 | ≥64 | ≥64 | ≥16 | ≥16 | ≥16 | ≥64 | ≥4 | 1 | **-** | **-** | **+** | **+** | **+** | **-** | **-** | VIM, KPC | **A1** | |  |
| ED01498924 | *A. baumannii* | Tertiary | Surgery | Tissue | ≥32 | ≥64 | ≥64 | ≥64 | ≥16 | ≥16 | ≥16 | ≥64 | ≥4 | ≤0.5 | **-** | **-** | **+** | **+** | **+** | **-** | **-** | VIM, KPC | **A1** | |  |
| ED01502268 | *K. pneumoniae* | Tertiary | Medicine | Sputum | ≥32 | ≤4 | ≥64 | ≥64 | ≤0.25 | ≤0.25 | ≥16 | 8 | ≥4 | 1 | **+** | **+** | **+** | **+** | **+** |  | **+** | - | **K1** | |  |
| ED01503757 | *K. pneumoniae* | Tertiary | Medicine | Sputum | ≥32 | ≤4 | ≥64 | ≥64 | ≤0.25 | ≤0.25 | ≥16 | 8 | ≥4 | 1 | **+** | **+** | **-** | **+** | **+** | **+** | **-** | - | K2 | |  |
| ED01501066 | *P. aeruginosa* | Tertiary | Urology clinic | Urine | ≥32 | ≥64 | 8 | 2 | ≤0.25 | 1 | 2 | 4 | ≤0.25 | ≥8 | **-** | **-** | **-** | **+** | **+** | **-** | **-** | - | P2 | |  |
| ED01504366 | *P. aeruginosa* | Tertiary | Surgery | Suppuration | ≥32 | ≥64 | 32 | 4 | ≤0.25 | 2 | ≤2 | 2 | ≤0.25 | ≥8 | **-** | **-** | **-** | **+** | **+** | **-** | **-** | - | / | |  |
| ED01504363 | *K. pneumoniae* | Tertiary | Medicine | Suppuration | ≥32 | 8 | ≥64 | 32 | 8 | ≤0.25 | ≥16 | 4 | ≥4 | ≤0.5 | **-** | **-** | **+** | **+** | **+** | **-** | **-** | VIM, KPC | **K3** | |  |
| ED01507915 | *P. aeruginosa* | Tertiary | ICU | Suppuration | ≥32 | ≥64 | ≥64 | ≥64 | 1 | 1 | ≤1 | ≤2 | ≤0.25 | ≥8 | **-** | **-** | **-** | **+** | **+** | **-** | **-** | - | P1 | |  |
| ED01507331 | *P. aeruginosa* | Tertiary | Surgery | Suppuration | ≥32 | ≥64 | 32 | 4 | 1 | ≤0.25 | ≤1 | ≤2 | ≤0.25 | ≥8 | **-** | **-** | **-** | **+** | **+** | **-** | **-** | - | / | |  |
| ED01507028-1 | *P. aeruginosa* | Tertiary | ICU | Tracheal aspirate | ≥32 | ≥64 | 32 | 4 | 1 | 1 | ≥16 | 16 | ≤0.25 | ≥8 | **-** | **-** | **-** | **+** | **+** | **-** | **-** | - | P1 | |  |
| ED01506083 | *A. baumannii* | Tertiary | Obstetrics and gynecology | Catheter tip | ≥32 | ≥64 | ≥64 | ≥64 | ≥16 | ≥16 | ≥16 | ≤2 | ≥4 | 4 | **-** | **-** | **-** | **+** | **+** | **-** | **-** | KPC | A1 | |  |
| ED01506443 | *A. baumannii* | Tertiary | Surgery | Catheter tip | ≥32 | ≥64 | ≥64 | ≥64 | ≥16 | ≥16 | ≥16 | ≥64 | ≥4 | 2 | **-** | **-** | **-** | **+** | **+** | **-** | **-** | KPC | A1 | |  |
| ED01506571 | *A. baumannii* | Tertiary | ICU | Suppuration | ≥32 | ≥64 | ≥64 | 16 | ≥16 | ≥16 | ≥16 | ≤2 | ≥4 | 1 | **-** | **-** | **-** | **+** | **+** | **-** | **-** | VIM, KPC | A2 | |  |
| ED01507028-2 | *A. baumannii* | Tertiary | ICU | Tracheal aspirate | ≥32 | ≥64 | ≥64 | 16 | ≥16 | ≥16 | ≥16 | ≥64 | ≥4 | 4 | **+** | **-** | **-** | **+** | **+** | **-** | **-** | - | A2 | |  |
| ED01506433 | *P. aeruginosa* | Tertiary | ICU | Wound | ≥32 | ≥64 | ≥64 | 4 | 2 | ≤0.25 | ≤1 | ≤2 | ≤0.25 | ≥8 | **-** | **-** | **-** | **+** | **+** | **-** | **-** | **-** | **/** | |  |
